# Dynamics and characteristics of misinformation related to earthquake predictions on Twitter

**DOI:** 10.1038/s41598-023-40399-9

**Published:** 2023-08-17

**Authors:** Irina Dallo, Or Elroy, Laure Fallou, Nadejda Komendantova, Abraham Yosipof

**Affiliations:** 1 Swiss Seismological Service at ETH Zurich, ETH Zurich, Sonnegstrasse 5, 8092 Zurich, Switzerland; 2 Faculty of Information Systems and Computer Science, College of Law and Business, Ramat-Gan, Israel; 3 Centre Sismologique Euro-Méditerranéen/Euro-Mediterranean Seismological Centre, Arpajon Cedex, France; 4 International Institute for Applied Systems Analysis, Laxenburg, Austria

**Keywords:** Seismology, Natural hazards

## Abstract

The spread of misinformation on social media can lead to inappropriate behaviors that can make disasters worse. In our study, we focused on tweets containing misinformation about earthquake predictions and analyzed their dynamics. To this end, we retrieved 82,129 tweets over a period of 2 years (March 2020–March 2022) and hand-labeled 4157 tweets. We used RoBERTa to classify the complete dataset and analyzed the results. We found that (1) there are significantly more not-misinformation than misinformation tweets; (2) earthquake predictions are continuously present on Twitter with peaks after felt events; and (3) prediction misinformation tweets sometimes link or tag official earthquake notifications from credible sources. These insights indicate that official institutions present on social media should continuously address misinformation (even in quiet times when no event occurred), check that their institution is not tagged/linked in misinformation tweets, and provide authoritative sources that can be used to support their arguments against unfounded earthquake predictions.

## Introduction

Recent studies have shown that myths about earthquakes are omnipresent in societies and that the claims and predictions made about these events can exacerbate them^1^. For example, after the Mw 5.6 Albanian earthquake in 2019, fake news about a possible magnitude 6.0 aftershock started to spread, leading people to flee the city in panic^2^. Other earthquake events have been exacerbated by misinformation related to predictions: the 2018 Palu earthquake^3^, the 2017 Mexico earthquake^4^, and the false prediction of the New Madrid, Missouri, earthquake made by American businessman Iben Browning in 1990^5^. Further misconceptions related to earthquake prediction are that (1) small earthquakes prevent big ones from happening, whereas the L’Aquila earthquake sequence in 2009 shows that this is not a given^6^; (2) animals can predict earthquakes; or (3) humans can create earthquakes (e.g., the government can create earthquakes and thus know when they will happen)^7^.

However, earthquakes cannot be predicted, meaning that the exact location, time, and magnitude of the next large event cannot be specified. What scientists can do is provide a forecast, namely to estimate the probability of earthquakes of a certain magnitude occurring in each space–time magnitude domain^8^. This forecast can be for long-time horizons (e.g., decades or a century), which is the basis for the long-term hazard maps most countries have, or for short-term horizons (e.g., days to weeks). Short-term forecasts are currently publicly available in some countries such as New Zealand and the United States (USA)—the USA only provides aftershock forecasts after magnitude 5 or higher events. In comparison, earthquake forecasts in Italy are continuously communicated to civil protection but not to the public as there is no clear legal basis for doing so. In other countries, such as Switzerland, earthquake forecasting models have been developed and will be transferred into operational service in the years to come.

Precise and well-defined communication efforts are vital when earthquake forecasts are issued^9^. It is particularly important, however, for the institutions responsible for public communications to clarify that it is impossible to predict the precise location, time, and magnitude of earthquakes. The same applies to other technologies/services such as Earthquake Early Warnings (EEW)^10,11^; in such cases, the public struggles to grasp that warnings can only be issued after the detection of P-waves, which indicate that the earthquake has already occurred, and relate to the arriving (destructive) S-waves. Responsible institutions (e.g., seismological services, public authorities) must be prepared to fight the spread of misinformation, especially after severe events, when people are anxious, in shock or panic, and, thus more prone to believing misinformation^12^.

On social media especially, misinformation can spread around the world in a few seconds through, for example, automated software^13^. Self-proclaimed experts, who believe they can predict the precise location and time of an earthquake, can benefit from this fast distribution, as it allows them to reach a wide audience. Furthermore, a social media platform like Twitter prioritizes user engagement over accuracy^14^, which makes it difficult to minimize the spread of fake news. Moreover, misinformation messages are often compelling narratives that are very appealing to people or trigger emotions^15^, increasing people’s desire to share them. Thus, a better understanding is needed of who spreads misinformation and in what sorts of patterns to counteract the effects of such Tweets (“Misinformation on Twitter”); it is only then that the institutions responsible for communicating information about earthquakes can adjust their strategies to the specific dynamics that occur on social media^11,16^.

In recent years, various studies modeled and analyzed the characteristics of fake news and misinformation in tweets. The spread and propagation of fake news were investigated by a few research papers^17,18^. Murayama et al.^17^ proposed a time-dependent model for the information spread of fake news on Twitter. The model uses retweets of specific news stories to predict the spread of fake news. The model suffers from several drawbacks. The model requires relatively high minimum of 300 posts on the fake news, over a relatively long period of at least 36 h. Zhao et al.^18^ further used the re-posting relationship between different users to establish a propagation network and found that fake news presents different topological features from real news.

In this research, we analyzed misinformation related to the subject of earthquake prediction, which is almost exclusively driven by discrete posts with a low number of retweets. The user network is therefore very small, mostly not related to specific stories, and of short duration. Considering the substantially different characteristics, the methodologies presented in previous research by Murayama et al.^17^ and Zhao et al.^18^ are therefore not suitable for the task.

In the context of the COVID-19 pandemic, Erokhin et al.^19^ analyzed the tweets frequencies of COVID-19 conspiracy theories and performed time-series analyses to identify patterns and categorize them into groups. Elroy and Yosipof^20^ used BERT to classify tweets as supporters and opponents of the COVID-19 5G conspiracy theory and analyzed the results. The results showed that supporters of the conspiracy theory use URLs in their tweets more often than opponents of the conspiracy theory. Further, there are substantially more tweets from supporters of a conspiracy when it first emerges, followed by a sharp decrease. Other studies that inspected different hazards, concluded that tweets are more emotionally laden and likely to be shared^13^.

However, not all emergencies are equal. Earthquakes and epidemics are emergency situations that present different features and risks. The risk perception is therefore different, which may consequently affect misinformation and its various features (e.g., frequency, spread, retweets). This research thus analyzes the dynamics and patterns of tweets related to earthquake prediction. To the best of our knowledge, our study is the first to collect, classify, and analyze all English-language tweets within the search criteria to check for misinformation related to earthquake predictions over a period of 2 years.

In total, we collected and analyzed 82,129 tweets related to earthquake predictions from 1 March 2020 to 31 March 2022. This allowed us (1) to analyze the fluctuations in earthquake prediction tweets over time and their characteristics, and (2) to compare the prediction tweets with the frequency of general earthquake notifications and with the tweets that were specifically trying to counteract earthquake prediction misinformation. Our study is thus a global meta-analysis of English-language tweets about earthquake prediction misinformation. More precisely, we answered the following questions:

1. What are the dynamics and frequencies of earthquake prediction misinformation tweets and not-misinformation tweets (over time)? [RQ1]
2. What are the characteristic differences between earthquake prediction misinformation tweets and not-misinformation tweets? [RQ2]
3. What are the characteristic differences between (1) users who spread earthquake prediction misinformation, (2) users who tweet accurate earthquake notifications, and (3) users who actively counter earthquake misinformation? [RQ3]
4. What are the differences between earthquake prediction misinformation and not-misinformation tweets regarding the usage of media and URLs? [RQ4]

The insights from our study are helping communication experts to better understand the dynamics of earthquake predictions on social media and, consequently, to adjust their communication efforts to counteract misinformation.

## Misinformation on Twitter

### Misinformation in general

Misinformation is false information that is judged, according to the best available evidence at the time, to be correct; and it is spread without any intention to deceive. In comparison, disinformation is false information spread deliberately to deceive people^21^. As it is not always possible to clearly distinguish between mis- and disinformation, we henceforth use the term misinformation to cover both.

Misinformation comes in different shapes and forms, from mere rumors and gossip, through conspiracy theories, to deliberate fake news. Misinformation can spread quickly around the globe and lead to behavioral patterns that make an emergency worse, as evidenced in various disasters such as the COVID-19 pandemic^22,23^, earthquakes^3^, and tsunamis^24^. Mis- and disinformation have in common various inherent qualities that facilitate their spread; these include uncertainties^12^, lack of authoritative information^25,26^, lack of science and information literacy^14^, and lack of sense-making processes to understand what is happening^27^. One study even showed that conspiracies vary across different platforms^28^ because people interact on platforms that best reflect their sense of self.

The spread of misinformation can trigger fear and anxiety, as shown after severe earthquakes^22^ and during health emergencies^23^. Moreover, misinformation can create unnecessary panic; during the 2018 floods in the state of Kerala in South India, for example, a fake video on water leakage from the Mullaperyar Dam created panic among the citizens, negatively impacting the rescue operations^29^. Spread of misinformation can further lead to unnecessary evacuations, such as when, during the 2018 earthquake in Hokkaido (Japan), unaffected communities were encouraged by rumors to evacuate^30^. This can lead to additional challenges and fewer resources being available for the communities that are affected. It can also pose a considerable threat to public health^31^, as well as causing fatalities that could have been avoided^32^.

There are, however, various strategies that responsible communication experts can use to counteract the spread of misinformation. “Prebunking” is a psychological inoculation strategy whereby the public is forewarned about the possibility of being misled by misinformation, and then later exposed to small doses of misinformation along with strong countering statements^30^. The goal is to make the public more resistant to false information before they are exposed to it^33^. Another more technical option is to correct messages using software robots (bots). Fake news bots aim to classify information on social media and inform users about possible fake news^4^ by using corrective algorithms, keywords, and hashtags^34^. Ozturk et al.^35^ showed that warnings such as “this posting may contain misinformation” can decrease users’ willingness to repost it. Further, people should be given the tools to question content and its source^32^, provided with reliable information sources^36^, and supported by correctly interpreting scientific evidence/information^37^. In addition to these strategies, reliable communication needs to include and address societies’ needs, concerns, and attitudes in order to increase its relevance^9^.

### Dynamics on social media

On social media, it is not only individuals and private entities that communicate and share information—online communities have also emerged in the last decades. These communities play a crucial role in, among other things, supporting citizens and intervention agencies in humanitarian aid distribution^38^. Social media has also been used to perform vital relief functions, provide information on damage, support disabled individuals, and offer moral support systems^24^. In the absence of authoritative statements, social media platforms can also help communities to handle emergencies themselves^25^.

However, certain dynamics on social media foster the spread of misinformation. For example, algorithmically curated social media platforms such as Twitter prioritize user engagement over accuracy^14^, thus making the sharing of emotionally charged content more likely. Bots in particular are “super-spreaders” of misinformation because they can retweet articles just seconds after they appear and often use low-credibility sources^13^. Furthermore, programs create fake accounts (e.g., Internet Research Agency), which can influence public opinion, as was the case during the U.S. Presidential Election in 2015^39^. Another study has shown that fake news is about 70% more likely to be shared and reaches 1500 people six times faster than true information^40^; this is line with the results for misinformation videos about COVID-19 vaccines on YouTube^41^. For event-driven misinformation or conspiracies, however, it seems that discussions often dissipate quite quickly over time^20^.

Moreover, people may unintentionally spread misinformation because they do not pay enough attention to the content they are reading^31^, which may explain why users over the age of 65 spread more articles from fake news domains than the younger age groups^42^. Spreading misinformation does not, however, imply that people also believe it to be true^43^.

Regarding the messages themselves, it has been shown that more misinformation tweets related to the conspiracy about the link between 5G and the spread of COVID contain URLs than not-misinformation tweets do^20^, which makes the arguments they put forth more credible to those who read them. Misinformation tweets also tend to be written in more ambiguous language^23^, which leaves room for interpretation. In addition, conspiracies are compelling narratives; thus simple language, videos, and images are used that trigger emotions and are more relatable than scientific tweets^15,44^. People who deliberately share fake news and conspiracy theories use more negative emotions, anger words, and anxiety links, and refer to topics such as death, religion, and power^45^.

## Methodology

A primary challenge involved in analyzing misinformation in tweets is to classify many of them in a reliable fashion. Previous work attempted to use various tweet characteristics for this purpose with different levels of success. Beskow and Carley^46^ used characteristics of the tweets’ authors, such as the number of followers, as an indication of whether or not the author was a bot. Odonovan et al.^47^ and Gupta et al.^48^ found that features such as inclusion of URLs, mentions of other users, number of retweets, and the length of the tweet, can contribute to the credibility assessment of a tweet.

Developments in the field of Natural Language Processing (NLP) have introduced new algorithms and methods for text embedding and classification, such as the Bidirectional Encoder Representations from Transformers (BERT)^49^. BERT was found to provide superior results for different NLP tasks, including word embedding and sequence classification^50,51^. Schütz et al.^52^ experimented with multiple deep learning models, mostly variants of BERT, to detect fake news, and concluded that BERT transformers are a promising approach, as they achieve notable results even when using smaller datasets.

Several research papers have used NLP approaches, including BERT, to investigate tweets related to COVID-19 misinformation and conspiracy theories^20,53,54^. Batzdorfer et al.^53^ investigated the motifs and dynamics of COVID-19 conspiracy theory tweets by comparing tweets from a group of users that discussed a conspiracy and a group that did not. Micallef et al.^54^ used BERT embeddings to investigate and counter misinformation in tweets related to COVID-19 over a period of 5 months. Elroy and Yosipof^20^ used combinations of a Covid-Twitter-BERT^55^, a BERT model that was pre-trained on COVID-19-related data, with transformation to sentence embedding using Sentence BERT^56^, in order to classify 5G conspiracy tweets as opposing or supporting the COVID-19 5G conspiracy.

The Robustly Optimized BERT Pretraining Approach (RoBERTa) pre-trained BERT using different design decisions, leading to improved performance and state-of-the-art results^57–59^. Our study is the first to use RoBERTa to classify tweets regarding earthquake misinformation or not-misinformation.

### Workflow

**Figure 1:**
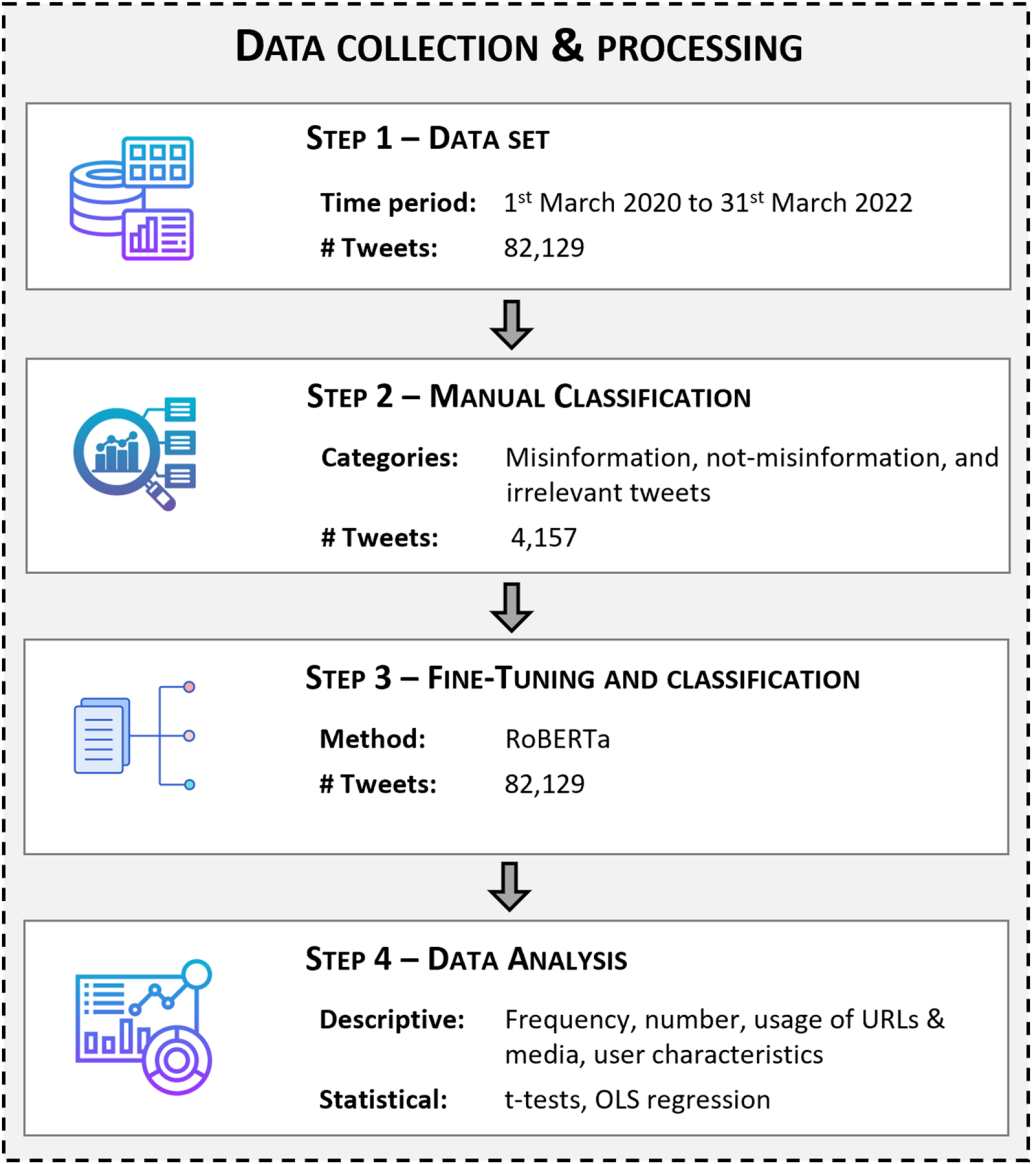
Overview of the data collection, fine-tuning, processing, and analysis.

The data processing and analyses consist of four steps, as depicted in Fig. 1 and described in the following sections.

#### Step 1: Dataset

The data was collected using the full search endpoint of Twitter API’s v2, which is limited to academic research and provides access to the full Twitter archive through the use of a search query. The search query retrieves tweets matching the specified criteria throughout the full archive. In our study, we used the search query [[predict OR forecast OR warn OR updates OR alert] AND [earthquake OR quake OR [seismic AND event] OR seismicity OR shaking OR EQ]]. With this query, we collected 82,129 tweets related to the subject of earthquake prediction, forecasts, and notifications, and the metadata of the users that posted the tweets, over a period of 2 years from 1 March 2020 to 31 March 2022. This study focused on both misinformation and not-misinformation to evaluate the scale of misinformation and avoid a biased impression that social media is rife with misinformation^43^.

#### Step 2: Manual classification

We manually labeled a sample of 4157 tweets into three categories: misinformation, not-misinformation, and irrelevant tweets. The labeling was performed separately by two social scientists working on hazard and risk communication in the field of seismology. The tweets were selected randomly. Disagreements between the classifications were resolved by a third person. The Cohen’s kappa coefficient of κ = 0.995 for the classified dataset shows an almost perfect level of agreement^60^.

The misinformation category includes all tweets that claimed to be able to predict a future earthquake, according to the state-of-the-art summarized in the recently published Communication Guide^1^. The not-misinformation category includes general earthquake notifications, tweets clarifying that earthquakes cannot be predicted, and tweets explaining what information is available and how certain services work (e.g., earthquake forecasting, EEW). All other tweets, such as tweets unrelated to earthquakes and discussions about secondary hazards triggered by earthquakes, were classified as irrelevant.

To avoid bias in the model when fine-tuning, we applied a preprocessing methodology similar to Nguyen et al.^61^ and removed tweets that were identical or very similar to each other from the manually labeled dataset. We limited the dataset to tweets that were 350 characters or shorter after preprocessing, thus removing a negligible 0.5% of the manually labeled dataset and reducing the maximum length of tweets by about 50%. The final data comprises 81,862 tweets, of which 3584 were manually labeled. The labeled dataset has 698 misinformation tweets, 1328 not-misinformation tweets, and 1558 irrelevant tweets. Table 1 presents the categories, sample tweets, and the number and percentage of tweets in each category.

**Table 1:** Examples and number of manually labeled tweets in each category.

Category	Example tweets	#Tweets	% Tweets
Misinformation	“24 HOUR WARNING: 5.5 + earthquake is likely in the Mammoth Lakes-Bridgeport area and 5.0 + earthquake is likely within 50 miles of Santa Clarita-NW of Los Angeles during the next 24 h” “Earthquakes: One more set of earthquake forecasts came true. All places, magnitudes and dates were in the predicted range. Magnitude range wr 5.1–6.3 (predicted wr 4.8–5.9) except *[@mentions]* (4.5)”	698	19.5
Not-misinformation	“No one can accurately predict earthquakes. The USGS issues long-term earthquake forecasts for certain areas” “Via @USGS_Quakes February is Earthquake Awareness Month. Here’s an EQ FAQ: Can you predict earthquakes? Nope. We can only calculate the probability that a significant earthquake will occur in a specific area within a certain number of years *[link]*”	1328	37.1
Irrelevant	“Could end in 5 billion gallons of lava or nothing will happen. Hard to say *[link]*”	1558	43.4

#### Step 3: Fine-tuning and classification

Fine-tuning is the process of adapting the pre-trained RoBERTa model to a specific task or domain by updating its parameters with a small amount of labeled data. Fine-tuning typically requires the splitting of the data into three sets, namely a training set, a validation set, and a test set. The training set is used to update the parameters of the model, and the validation set is continuously used during the training process to evaluate the model. Once the training is complete, the fine-tuned model is tested on the test set.

We used two steps to fine-tune the RoBERTa model. First, we used fivefold cross validation to confirm the viability of the model over different splits of the data. Second, after establishing the model as viable, we were able to fine-tune a model without using a test set, therefore maximizing the data available for training.

First, we fine-tuned a RoBERTa-base model using the labeled dataset five times for 10 epochs, each with a dropout of 0.2, weight decay of 0.01, learning rate of 1e-5, and a batch size of 16, using stratified fivefold cross validation on the labeled tweets. The test set in each fold was split into 10% for the validation set and 10% for the test set. The models converged at epoch 5 with an average evaluation loss of 0.446 ± 0.042 and an average F1 score of 0.846 ± 0.018 on the validation set.

The results for the test set at epoch 5 were evaluated using weighted F1, precision, and recall scores. The average F1, precision, and recall scores of the five models on the respective test sets were 0.845 ± 0.019, 0.85 ± 0.016, and 0.85 ± 0.016, respectively. These results show that the model is viable.

Second, for the final classification model of the data, we fine-tuned a RoBERTa model once more using 90% of the labeled dataset for the training set and 10% for the validation set, without a test set. Considering the model had already been established as viable, this approach enabled the use of a larger dataset for training and fine-tuning. The model converged at epoch 4 with a loss of 0.3574, an evaluation loss of 0.4919, and an F1 of 0.8494 on the validation set.

The fine-tuned model was used for the classification of the complete dataset into one of three categories. The results of the classification of the full data set with the fine-tuned model are described in Table 2.

**Table 2:** Number of tweets, users, and tweets per user in each category.

Category	# of tweets	# of users	Tweets/user
Misinformation	9857	3804	2.59
Not-misinformation	35,056	4931	7.11
Irrelevant	36,949	27,118	1.36
Total	81,862	34,052	2.40

#### Step 4: Analysis

To answer our research questions, we conducted a descriptive analysis of the frequency of misinformation and not-misinformation tweets (over time). We used an ordinary least squares (OLS) time series model to analyze the effect of not-misinformation tweets on the spread of misinformation tweets and vice versa. Additionally, to answer RQ2 and RQ3, we used a statistically independent sample t-test to compare the tweets and the users in the not-misinformation and misinformation groups. We tested the differences between the tweets in four variables: number of likes, number of replies, number of retweets, and sentiment of the tweet. For the user group comparison, we used the following four variables: number of tweets per user, number of followers per user, number of other users a user is following, and user’s age on the platform in days.

## Results and discussion

The results are structured along the four research questions: frequency (over time) (RQ1); tweet and user characteristics (RQ2 and RQ3); and usage of media and URL (RQ4). The results allowed us to derive recommendations for institutions trying to minimize and fight the spread of, and belief in, misinformation, as described in “Discussion”.

### Frequency (over time)

#### Frequency of not-misinformation and misinformation tweets

In total, 82,129 tweets were considered for the analysis and classified into three categories, as described in Table 2: 39,266 were found to be irrelevant, that is, they did not refer to earthquakes or information about secondary hazards triggered by earthquakes (e.g., power grid damage). This relatively high number of irrelevant tweets is a consequence of the search criteria chosen, which provide wide coverage of the discussion.

The remaining 34,321 tweets—classified as not-misinformation—were primarily general earthquake notifications from official sources providing information about the location, time, and affected area of an event that had occurred. A small part of these tweets were specific tweets clarifying that earthquakes cannot be predicted.

Especially in the context of EEW-related tweets, it was explained that predictions are not possible and that EEW alerts are sent after an earthquake has been detected: “*EEW systems cannot predict earthquakes, but they can provide up to tens of seconds of warning by detecting an earthquake immediately after it occurs*”. National “Earthquake Preparedness Days” were also used to educate people about earthquake facts and misinformation: *“For an earthquake prediction to be meaningful, it must specify a time, location, and magnitude range that is unlikely to occur randomly.—Who can predict an earthquake? (Spoiler: no one)*”. Such dedicated days are always an opportunity to sensitize people to hazards and risks, and to clarify what is correct and what is not, in alignment with the prebunking strategy^31^.

Overall, there are substantially more not-misinformation tweets than misinformation tweets, which indicates that accurate and reliable information dominates the Twitter environment. There are also more users in the not-misinformation group, and those users tweet more (7.11 tweets per user) than the users in the misinformation group (2.59 tweets per user), as described in Table 2.

#### Temporal dynamics

**Figure 2:**
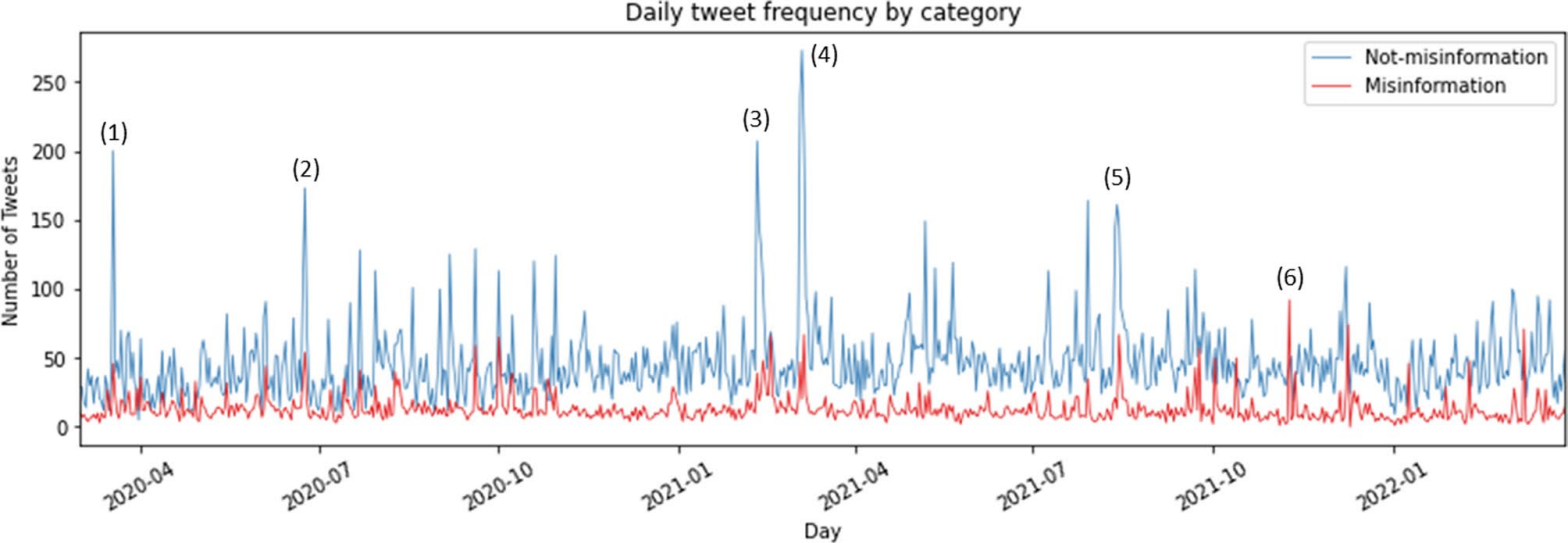
Daily frequency of tweets for both categories. The peaks represent discussions regarding the following earthquakes: (1) 18 March 2020, Mw 5.7, Salt Lake City, Utah, USA; (2) 23 June 2020, Mw 7.4, Mexico; (3) 10 February 2021, Mw 7.7, Loyalty Islands; (4) 4 March 2021, Mw 8.1, Keramedac Islands; (5) 14 August 2021, Mw 7.2, Haiti; (6) 9 November 2021, anti-forecast peak.

We further analyzed the frequency over time of both “misinformation” and “not-misinformation” tweets. Figure 2 presents the daily frequency of the tweets per category, with clear peaks and fluctuations being visible. The daily peaks of the two categories (misinformation and not-misinformation) often correlate, showing that after a major event and during earthquake sequences the spread of predictions increased.

For instance, the two highest peaks, on 10 February 2021 and 4 March 2021 (Fig. 2, annotations 3–4) show that high-magnitude earthquakes triggering aftershocks of relatively high magnitudes receive high attention internationally. On 10 February 2021, when a Mw 7.7 thrust earthquake ruptured the megathrust along the southeast Loyalty Islands (New Caledonia)^62^, 217 general earthquake notifications about the event were published on Twitter. This earthquake sequence led to 25 tweets that claimed to predict the next megathrust earthquake in or near this area.

The highest peak in the period of our data sample was on 4 March 2021 when the Keramedac Islands (part of New Zealand’s outlying islands) experienced two major shocks, namely a Mw 7.4 foreshock and a Mw 8.1 main shock^63^. After the event, people on Twitter either stated that they predicted the earthquake: *“And there you go. Big earthquake just as I predicted 4 days ago. Why am I the only one who seems to know how to predict earthquakes. It has all to do with the solar cycles”* or claimed that they can predict the next one: *“… Yep, an earthquake almost 4000 miles away. If we get anything, the first one is expected at 4:35 this afternoon. Weird how they can predict the time so precisely”.* Claims about animals having predicted the earthquake were also observed, such as: *“Dogs warn owners of early-morning 7.1 earthquake and tsunami risk on East Coast …”.*

The other peaks represent other earthquakes with a lower magnitude: (1) tweets on 18 March 2020 (Fig. 2, annotation 1) were mainly about the Mw 5.8 earthquake in Salt Lake City, Utah, USA, which was the strongest earthquake in the region for the last 28 years. Some people wrote that others should stop claiming to have the ability to predict an even larger further earthquake, as predictions are not possible: *“Stop tweeting and/or retweeting rumors about a bigger quake being imminent. You can’t predict earthquakes, dummies …”;* (2) On 23 June 2020 (Fig. 2, annotation 2), 9 km SE of Santa María Xadani, Mexico, a Mw 7.4 earthquake occurred with a tsunami alert for several regions. The quake caused damage to buildings, claimed 10 lives, and led to a multi-hazard threat as the following tweet highlights: *“We are at the point of highest contagion of Coronavirus. Then 7.1 earthquake this morning. Now we are on red alert for a possible tsunami. Mexico couldn't get any worse”*; (3) On 14 August 2021 (Fig. 2, annotation 5), a Mw 7.2 earthquake struck Haiti, triggering landslides, causing damage to several buildings, and claiming about 2000 fatalities and 12,000 injured. There were rumors that this earthquake had been predicted, as well as opposing voices denying any such prediction.

We further identified that increased discussions about past events on the anniversary day of their original occurrence are common. For instance, increased interest can be seen at the beginning of 2021 about the earthquake in Fukushima that occurred on 11 March 2011^64^. One exceptional peak in the misinformation group was found on 9 November 2021 (Fig. 2, annotation 6), where there was no recognizable relation to a specific earthquake event. On this date, a Twitter account published multiple earthquake anti-forecasts with statements about how unlikely it is for strong earthquakes to occur. These anti-forecasts were also often spread after low-magnitude events, where the account referenced the EMSC earthquake notification tweet and was linked to seismo.info.

To investigate the interaction between tweets in the misinformation and not-misinformation groups, we applied the OLS time series model (Eq. 1) to estimate the effect of the daily earthquake misinformation discussion (tweet frequency) on the not-misinformation discussion and vice-versa:

1 $${y}_{t}=a+{\beta }_{1}{x}_{t}+{\beta }_{2}{x}_{t-1}+{\beta }_{3}{x}_{t-7}{+\beta }_{4}{x}_{t-14}$$

For the misinformation OLS model, ${y}_{t}$ is the number of misinformation tweets on day t. ${x}_{t}$ is the number of tweets related to not-misinformation on day t, and ${x}_{t-1}$ is the number of tweets related to not-misinformation on day *t-1* (i.e., a day before). ${x}_{t-7}$ is the number of tweets related to not-misinformation on day *t-7* (i.e., a week before), and ${x}_{t-14}$ is the number of tweets related to not-misinformation on day $t-14$ (i.e., 2 weeks before). The not-misinformation OLS model takes the same form.

Table 3 summarizes the regression results. Model 1 is an OLS estimation for the misinformation discussion at time *t*. The results for model 1 indicate that the not-misinformation tweet frequency at time *t* is a positive and significant predictor of misinformation with a coefficient of 0.16. Model 2 is an OLS estimation for the not-misinformation discussion at time *t*. The results indicate that the misinformation tweet frequency at time *t* is a positive and significant predictor with a coefficient of 1.25. The results of the OLS models indicate that the tweet frequency dynamics over time of the misinformation and not-misinformation discussion share a simultaneous bidirectional effect.

**Table 3:** OLS regression results for the misinformation and not-misinformation tweets.

	Model 1 Misinformation_t_	Model 2 Not-misinformation_t_
Constant	5.27***	28.84***
Misinformation_t_		1.25***
Misinformation_t-1_		0.11
Misinformation_t-7_		− 0.05
Misinformation_t-14_		− 0.04
Not-misinformation_t_	0.16***	
Not-misinformation_t-1_	0.02	
Not-misinformation_t-7_	0	
Not-misinformation_t-14_	− 0.01	
N	746	746
R^2^	0.214	0.212

***p < 0.001, **p < 0.01, *p < 0.05.

### Characteristics of the tweets and users

Table 4 presents the differences between the tweets in both groups using the number of retweets, likes, replies, and sentiment score of each tweet. A comparison of the tweets in the not-misinformation and misinformation groups indicates that the mean sentiment score of tweets in the not-misinformation group (0.17 ± 0.27) is significantly higher (t(44,911) = 42.27, p value < 0.001) than the mean sentiment score in the misinformation group (0.03 ± 0.34).

**Table 4:** Independent sample t-test between the tweets in the not-misinformation and misinformation groups.

Feature	Not-misinformation (Mean ± SD)	Misinformation (Mean ± SD)	t-test	DF
Retweets	2.45 ± 30.73	4.26 ± 23.17	− 5.43***	44,911
Likes	4.65 ± 45.35	9.71 ± 104.39	− 7.01***	44,911
Replies	0.24 ± 2.29	0.66 ± 4.66	− 12.46***	44,911
Sentiment	0.17 ± 0.27	0.03 ± 0.34	42.27***	44,911

***p < 0.001, **p < 0.01, *p < 0.05.

However, the mean number of retweets in the not-misinformation group (2.45 ± 30.73) is significantly lower (t(44,911) = − 5.43, p value < 0.001) than the mean number of retweets in the misinformation group (4.26 ± 23.17). The mean number of likes is also significantly lower (t(44,911) = − 7.01, p value < 0.001) in the not-misinformation group (4.65 ± 45.35) than in the misinformation group (9.71 ± 104.39). The mean number of replies in the not-misinformation group (0.24 ± 2.29) is also significantly lower (t(44,911) = − 12.46, p value < 0.001) than in the misinformation group (0.66 ± 4.66).

Table 5 presents the differences between the users in both groups using the number of tweets, followers, numbers of following, and the time in days the user has existed on Twitter. No significant differences were found in the mean number of total tweets posted on the platform by users in either group or in the mean number of users these users follow. The mean number of users that follow the users in the not-misinformation group (69,557.64 ± 1,049,574.74) is significantly higher (t(7,987) = 2.823, p value < 0.05) than in the misinformation group (16,405.63 ± 390,900.55).

**Table 5:** Independent sample t-test between the users in the not-misinformation and misinformation groups.

Feature	Not-misinformation (Mean ± SD)	Misinformation (Mean ± SD)	t-test	DF
Tweets	47,802.14 ± 122,054.78	43,999.03 ± 130,438.43	1.338	7987
Followers	69,557.64 ± 1,049,574.74	16,405.63 ± 390,900.55	2.823*	7987
Following	1689.47 ± 7429.71	1669.39 ± 5334.5	0.134	7987
Days since user creation	3102.63 ± 1586.51	2905.74 ± 1545.64	5.552***	7987

***p < 0.001, **p < 0.01, *p < 0.05.

However, the mean number of days since users in the not-misinformation group were created on the platform (3102.63 ± 1586.51) is significantly higher (t(7,987) = 5.552, p value < 0.001) than that of users in the misinformation group (2905.74 ± 1545.64).

### Usage of media and URLs in tweets

Looking at the use of media and URLs is relevant to determine how each group reinforces its stance in the debate on Twitter. We processed and analyzed the use of media and URLs in the tweets of both groups. URLs were reduced to their top-level names and some aliases of major websites were combined with the main domain (e.g., nytim.es and nytimes.com). Domains that did not provide any value to the analyses were ignored (e.g., twitter.com, URL-shortening services, content management platforms).

By investigating the number of tweets in each group that made use of media (i.e., images, videos, GIFs), we found that in both the not-misinformation and the misinformation group, only about 18–24% of the tweets in each group contained media, and no significant differences were found between the groups regarding the use of media in tweets.

**Figure 3:**
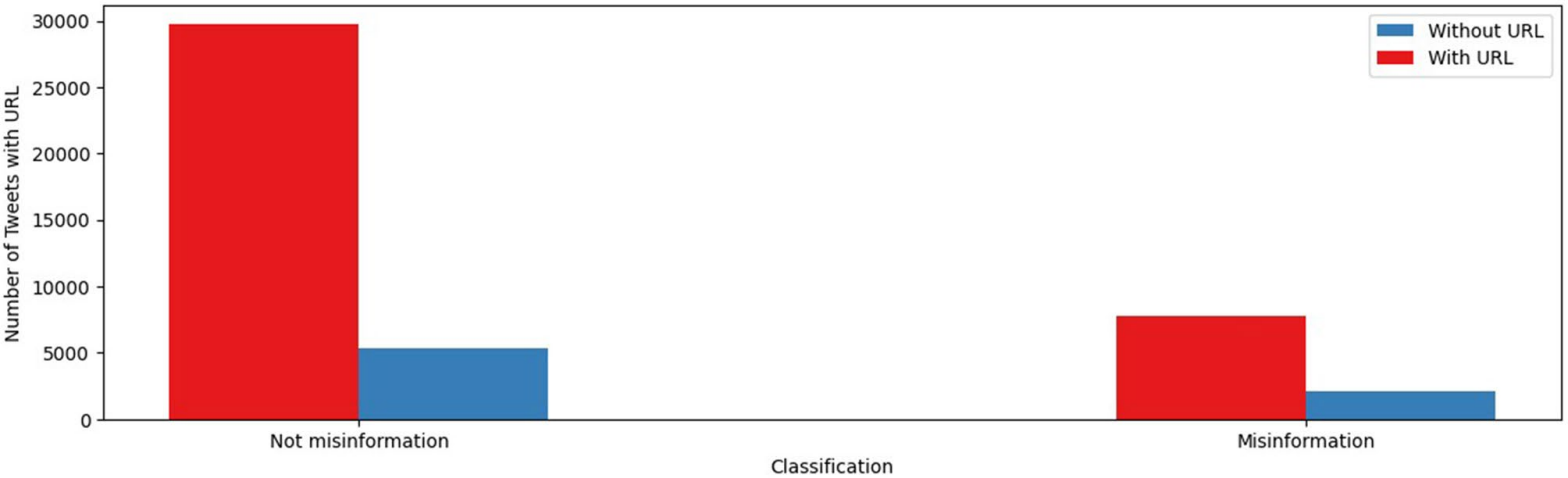
Use of URLs in the not-misinformation and misinformation tweets.

Regarding the use of URLs in tweets, we found that 79.1% and 84.8% of the tweets in the misinformation group and the not-misinformation group, respectively, contained one or more URLs. Figure 3 compares the number of tweets that linked at least one URL in each group.

**Figure 4:**
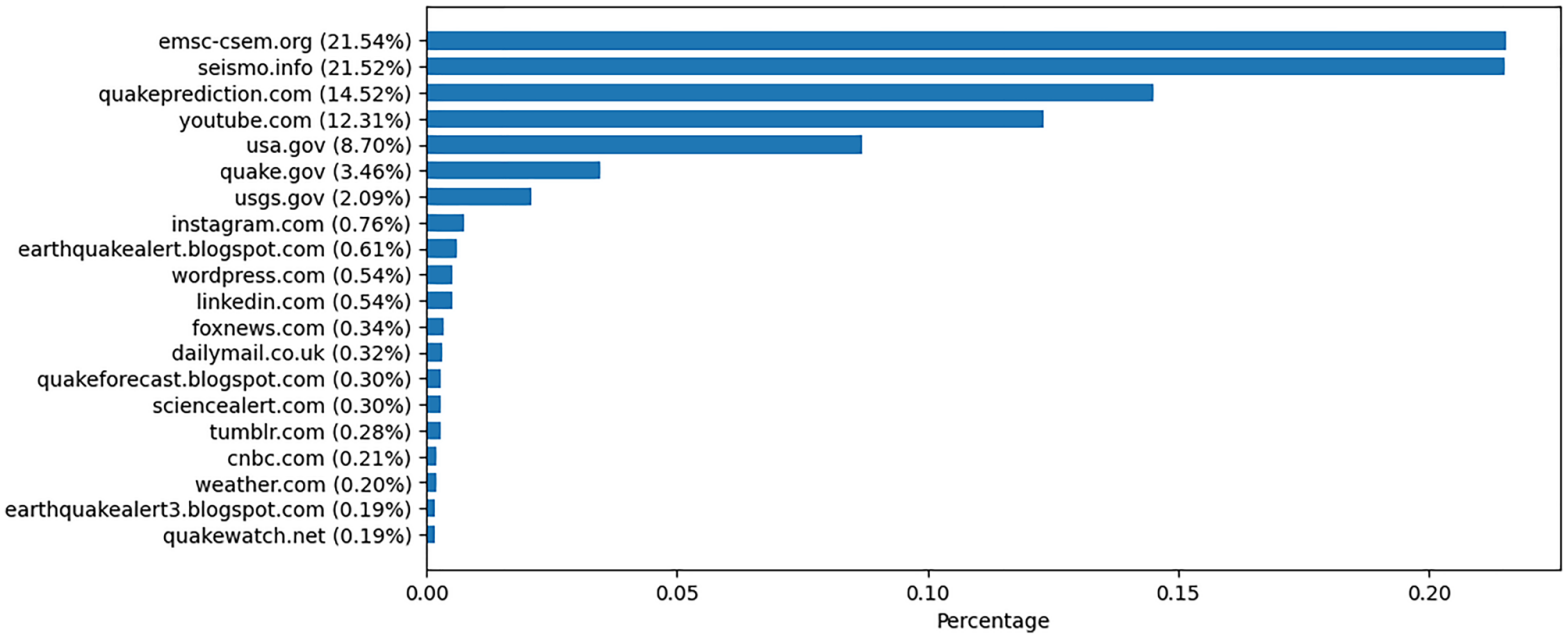
Percentage of the top 20 most linked domains in the misinformation tweets.

Figure 4 presents the most referenced URLs in the misinformation group. The results show that over 50% of the tweets linked to three websites, namely *emsc-csem.org* (21.5%), *seismo.info* (21.5%), and *quakeprediction.com* (14.5%). YouTube was linked in almost 12.3% of the tweets. *Seismo.info* and *quakeprediction.com* are privately run websites that claim to predict or forecast earthquakes. The website of the European Mediterranean Seismological Centre (EMSC) (*emsc-cesm.org*) was linked by an individual stating they could predict earthquakes, therefore wrongfully using accurate earthquake notifications. The individual in question refrained from that behavior following a request by the EMSC to stop referencing their earthquake notifications in tweets. This request was in line with EMSC policy to fight earthquake misinformation and to not be associated with it^11,16^.

**Figure 5:**
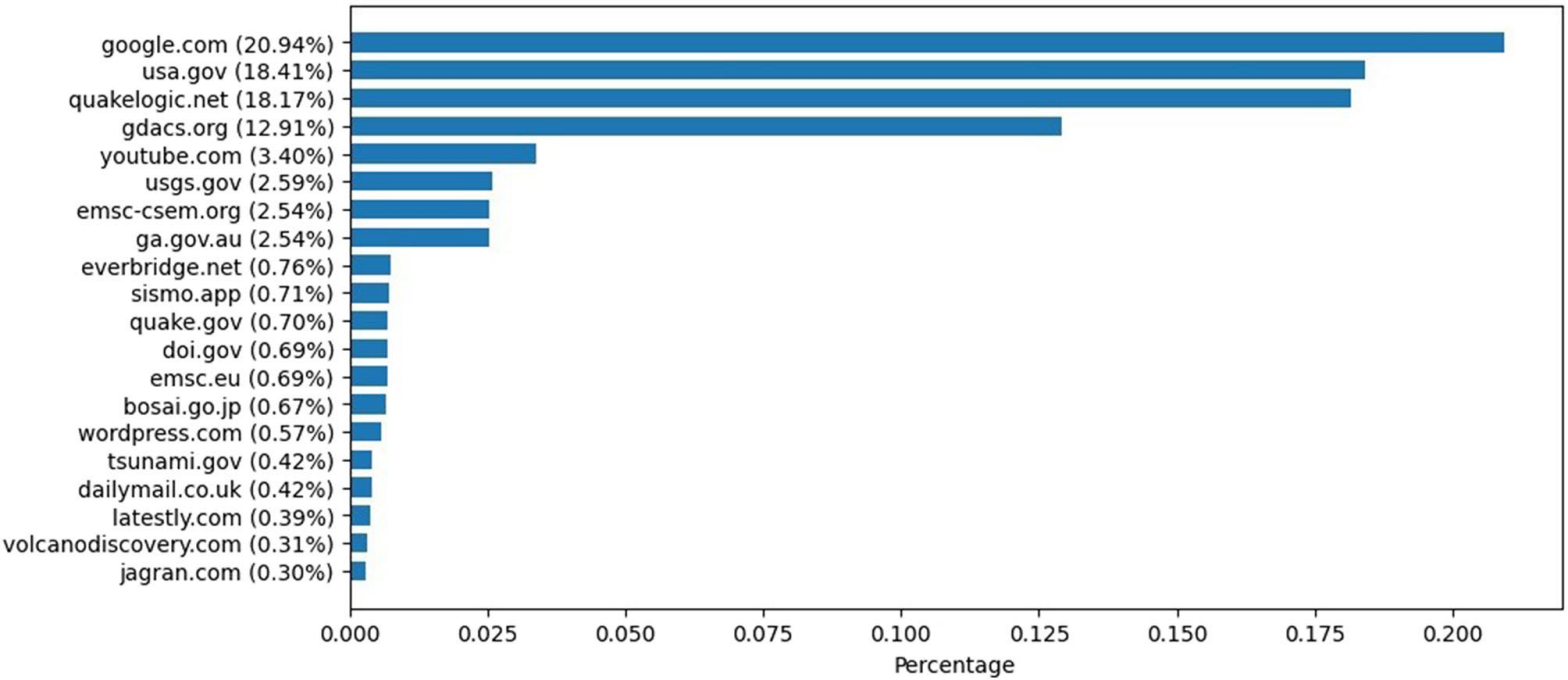
Percentage of the top 20 most linked domains in the not-misinformation tweets.

Figure 5 presents the most referenced URLs in the not-misinformation group. The results show that most of the URLs referenced in this group are of recognized, reliable reporting websites and official authorities such as usa.gov. Google was noticeably the most referenced top-level domain. Google itself, with some exceptions, generally refers to other sources of information, and the credibility relies on the credibility of the original source. A deeper analysis of the actual URLs linked under this top-level domain reveals that almost all the URLs link to specific locations on Google Maps. This finding, along with the lack of similar indications in the misinformation group, suggests that actual reports refer to specific geographical locations, whereas misinformation reports tend to be more ambiguous.

## Discussion

Our study is the first meta-analysis of global-level English-language tweets related to misinformation about earthquake predictions as compared with general earthquake notifications on a global level. It thus complements insights from analysis of local discourses that have already been conducted^2,4^. Table 6 provides an overview of our main insights (first column) and the derived recommendations for institutions, individuals, or groups in charge of, or willing to counteract, the spread of misinformation (second column). We argue that the recommendations derived from our meta-analysis can be transferred to local discourses impacted by earthquake predictions (Table 6), while of course considering contextual factors and the local environment^1^.

**Table 6:** The main insights from our study and recommendations on how to use these insights to counteract misinformation tweets.

Insights	Recommendations
*Earthquake predictions are continuously present on Twitter with peaks after felt earthquakes* The fact that earthquake predictions are continuously present on Twitter means that there is a continuous need for communication measures to counteract them, even in quiet times (e.g., campaigns, social events). However, it is particularly important after felt events, to devote attention and resources to actively counteracting the spread of false predictions	Foster measures to counteract misinformation, especially after an event, but also at other times
*Tweeters link earthquake notifications from official sources in their earthquake prediction claims* This is especially critical because the readers might trust the official source and thus also believe in the misinformation. Therefore, official (governmental) institutions and agencies should continuously check that their notifications are not linked or that their account is not tagged in misinformation tweets	Check who is sharing, tagging, and linking your posts and, ask not to be associated with misleading content
*URLs are used more than media (pictures & videos) in tweets* Not-misinformation tweets especially also contain a URL to the official website of the notification. This allows users to access further detailed information if they wish. It further enables users who want to actively counteract predictions to provide reliable sources for their arguments	Provide information sources opponents can use to support their arguments
*Besides the private earthquake prediction websites, misinformation videos from YouTube are often linked in tweets* The communication strategy should address misinformation on the different social media platforms, as the misinformation sources are also shared on the other platforms. Further, as the types of misinformation may vary across the platforms, different communication approaches are needed^28^	Monitor multiple social media channels
*Decision support tools are an essential part of every policy process* Decision support tools are a crucial component of an informed policy-making process. These resources come in a variety of shapes and sizes, including software applications, statistical models, tools for data visualization, and expert systems	Provide decision support tools to deal with misinformation on social media
*Misinformation, media policy, and critical thinking are all interrelated issues that have an impact on how people receive and understand information in the digital age* The term "media policy" refers to the rules and regulations that control the media sector, such as the laws governing ownership, free expression, and content management. The ability to evaluate facts, analyse information objectively, and reach well-founded conclusions is referred to as critical thinking	Create media policy measures to stimulate critical thinking
*Uncertainty can lead to anxiety, which in turn increases the chance that people will believe in misinformation* After an event, one of the first questions people ask is if this was the main shock or if a stronger earthquake will follow. In particular, when people receive the information that further earthquakes are expected, this uncertainty can lead to anxiety as the following tweet shows: “*This is what I’m worried about – that last night’s quake was a foreshock. I really hope not. They warned us last night about aftershocks for the next 7 days. They’ve been saying for years that a big one can strike at any time in the Kanto region.”* This anxiety in turn leads people more likely to believe in misinformation^22,65^ and can even trigger panic and inappropriate behaviors^2^	Treat emotions of users on social media seriously
*There were only a few tweets that actively clarified that it is impossible to predict an earthquake* As part of *National Earthquake Days*, some tweets sensitized people, in an attractive way, to the fact that earthquake predictions are not possible. During such dedicated days, people’s attention to learning something new and interest is heightened, improving the success of information campaigns^66^	Educate people when their attention is high (e.g., holding national or local earthquake days)
*An earthquake rarely comes alone* The events analyzed in this study show that strong earthquakes, in most cases, trigger secondary hazards that are an additional threat to the affected societies (e.g., tsunamis, landslides, volcanos). Further, people indicated that they were so frightened by COVID-19 that any further disaster/emergency was an additional burden, which can have negative psychological effects	Consider multi-hazard communication
*General notifications and correct information about earthquake (events) dominate* Our results show that general notification and information about dominate the debates on Twitter and people are thus coming across correct information with links to accurate official websites more often. This is important as misinformation then has less value and does not take over the discussion focus	Foster dissemination of correct information

In general, we identified that unlike an event-driven misinformation or conspiracy, such as those related to COVID-19 where the discussion dissipates over time^20^, earthquakes carry inherent uncertainty as they occur randomly, cannot be mitigated, and cannot be predicted, thus reigniting the discussion time after time. We also show, however, that after severe events, earthquake prediction claims are more often widespread on Twitter, which is problematic as affected people struggle to understand what is actually going on^27^. Especially after strong events, institutions responsible for communication with the public need to provide rapid, accurate information about the current situation so that people do not fill the information void with false information. This is in line with empirical observations made by the EMSC that served as a basis for developing a strategy to pre-bunk misinformation after significant earthquakes to prevent it from occurring^16^.

Regarding the elements used to support the statements of Twitter users, we identified that people on Twitter prefer to use URLs and not media elements to underscore their arguments. Unlike Elroy and Yosipof^20^ who showed that tweets related to misinformation on the connection between COVID-19 and 5G contain more URLs than tweets from opponents, we did not see this pattern for earthquake predictions. This could be explained by the fact that for the conspiracy *COVID-19 and 5G*, there was not much accurate information material available, whereas for earthquakes there is accurate information available, for example, on national seismological services’ information platforms, both immediately after an event and in quiet times. In line with other studies^45^, however, we did find that misinformation tweets contain more negative wording and foster more active interaction (e.g., number of replies). As not-misinformation tweets are more frequent than misinformation tweets about predictions, accurate information however dominates the debate. Further, the accounts providing correct earthquake information also have more followers, which shows that most Twitter users access accurate information.

## Conclusion

The internet and social media have recently made it simpler for misinformation to spread quickly and widely, and this has had important social and political repercussions. As a result, governments are taking action to address misinformation because of increasing concerns about how it is affecting the public debate. Inaccurate information can be especially destructive, as it can cause fatalities and increased property losses.

To lessen the effects of earthquakes, it is essential to have in place trustworthy earthquake notification systems and disaster risk reduction policies. This study offers insights into the dynamics of general earthquake notifications and misinformation messages related to earthquake predictions on Twitter. We evaluated the characteristics of these tweets and the accounts that published them. The recommendations we derived from these results should support (authoritative) institutions in counteracting misinformation on social media and also foster the spread of correct information. Fact-checking, media literacy, proper information transmission, reporting, and cooperation with social media platforms are all necessary components of a multi-pronged policy to counteract misinformation on social media. The transmission of misinformation on social media can be reduced if the actions recommended here to encourage the sharing of accurate information on social media are taken.

## Data Availability

The tweets dataset was collected from Twitter using a limited academic research API access. The dataset can be retrieved from Twitter according to the query explained in the methodology. Other data that support the results are available from the corresponding author upon reasonable request.
